# Instruments for assessing the risk of falls in acute hospitalized patients: a systematic review and meta-analysis

**DOI:** 10.1186/1472-6963-13-122

**Published:** 2013-04-02

**Authors:** Marta Aranda-Gallardo, Jose M Morales-Asencio, Jose C Canca-Sanchez, Silvia Barrero-Sojo, Claudia Perez-Jimenez, Angeles Morales-Fernandez, Margarita Enriquez de Luna-Rodriguez, Ana B Moya-Suarez, Ana M Mora-Banderas

**Affiliations:** 1 Department of Nursing, Agencia Sanitaria Costa del Sol, Autovia A7 km. 187, 29603, Marbella, Malaga, Spain; 2 Faculty of Health Sciences, University of Malaga, Malaga, Spain; 3 Quality of Health Care Unit, Agencia Sanitaria Costa del Sol, Autovia A7 km. 187, 29603, Marbella, Malaga, Spain

**Keywords:** Accidental falls, Adverse events, Clinical safety, Risk assessment, Inpatients, Systematic review, Nursing assessment

## Abstract

**Background:**

Falls are a serious problem for hospitalized patients, reducing the duration and quality of life. It is estimated that over 84% of all adverse events in hospitalized patients are related to falls. Some fall risk assessment tools have been developed and tested in environments other than those for which they were developed with serious validity discrepancies. The aim of this review is to determine the accuracy of instruments for detecting fall risk and predicting falls in acute hospitalized patients.

**Methods:**

Systematic review and meta-analysis. Main databases, related websites and grey literature were searched. Two blinded reviewers evaluated title and abstracts of the selected articles and, if they met inclusion criteria, methodological quality was assessed in a new blinded process. Meta-analyses of diagnostic ORs (DOR) and likelihood (LH) coefficients were performed with the random effects method. Forest plots were calculated for sensitivity and specificity, DOR and LH. Additionally, summary ROC (SROC) curves were calculated for every analysis.

**Results:**

Fourteen studies were selected for the review. The meta-analysis was performed with the Morse (MFS), STRATIFY and Hendrich II Fall Risk Model scales. The STRATIFY tool provided greater diagnostic validity, with a DOR value of 7.64 (4.86 - 12.00). A meta-regression was performed to assess the effect of average patient age over 65 years and the performance or otherwise of risk reassessments during the patient’s stay. The reassessment showed a significant reduction in the DOR on the MFS (rDOR 0.75, 95% CI: 0.64 - 0.89, p = 0.017).

**Conclusions:**

The STRATIFY scale was found to be the best tool for assessing the risk of falls by hospitalized acutely-ill adults. However, the behaviour of these instruments varies considerably depending on the population and the environment, and so their operation should be tested prior to implementation. Further studies are needed to investigate the effect of the reassessment of these instruments with respect to hospitalized adult patients, and to consider the real compliance by healthcare personnel with procedures related to patient safety, and in particular concerning the prevention of falls.

## Background

For hospitalized patients, falls are a serious problem, reducing the duration and quality of life. Older people with injuries have higher mortality rates and stay longer in hospital due to comorbidity. Falls are the predominant cause of injury in older people (over 65 years), followed by traffic accidents, fires and burns, drowning and poisoning. It has been reported that in the European Union there are 13.3-164.5 deaths per 100,000 persons among those older than 65 years [1].

It is estimated that over 84% of all adverse events in hospitalized patients are related to falls [2]. Approximately 30% of the hospitalized patients who fall suffer injuries, of which 4-6% are severe, including fractures, subdural haematomas, bleeding and even death [3].

The importance of this issue is such that the Joint Commission International (JCI) includes it among its safety standards in the accreditation manual for hospitals “Reduce the risk of patient harm resulting from falls” [4]. Furthermore, falls ranked sixth on the list of JCI sentinel events in 2012, with 477 notifications [5], a position among the most common adverse events it has held for the past three years [5].

As well as the physical consequences, there are also psychological ones, constituting what is known as the “post-fall syndrome”, which include fear of another fall, and the loss of self-esteem and independence, compromising the patient’s lifestyle and impacting on family caregivers.

The costs arising from falls, particularly hip fractures, skull fractures and leg injuries, represent a large proportion of healthcare spending. It is estimated that 92% of the costs of health care for patients who have suffered a fall are attributable to this factor [6], although it is difficult to obtain an accurate figure because most studies only include the costs of patients admitted following an injury, and do not take into account those who fall within the hospital itself [7]. An estimate by the British National Health Service estimated that about £15 million a year are incurred in hospital costs as a result of falls (£92,000 per year for an 800-bed hospital) [8].

Various studies have investigated the risk factors for falls in hospitals [3,9], identifying these as including advanced age, agitation, confusion or disorientation, generalized muscle and/or leg weakness, unstable gait, urinary incontinence, a history of previous falls, visual deficit or the use of certain medications (hypnotics, sedatives, vasodilators, diuretics, antidepressants, etc.) [3,9,10]. Moreover, the hospital environment itself can directly affect the incidence of falls. Extrinsic risk factors include the presence/absence of bed rails, the height and stability of any type of seat (including toilet) or obstacles in the form of clinical furniture and equipment [11]. The mere fact of hospitalisation represents a risk factor for falls. Older people especially can become more disoriented or agitated, or suffer diminished functionality during hospitalisation, and thus be at increased risk of falls [12].

Analysis of the circumstances in which falls occur among hospitalized acutely-ill patients and of the risk factors involved has led to the development of various instruments to assess the risk of falls, such as the Downton scale [13], the Morse Fall Scale (MFS) [14], the St. Thomas Risk Assessment Tool in falling elderly inpatients (STRATIFY) [15], the Tinetti test [16], the Conley scale [12], the Hendrich Fall Risk Model (HFRM) [17] and its later version HFRM II [18].

Some of these risk assessment tools have been tested in environments other than those for which they were developed [19-23], with disparate results, including difficulties for widespread use, serious validity discrepancies between the original authors’ version and successive ones [24], and in the heterogeneity of diagnostic accuracy in terms of cutoff points [22,25-27]. However, a recent Cochrane review showed that multifactorial interventions in hospitals reduce the rate of falls (rate ratio 0.69, 95% CI: 0.49 - 0.96), although risk assessment is addressed as one of many interventions, and it is not easy to isolate its specific effect [28]. Hospitalized patients in the acute phase of their disease have specific characteristics. Changes in acuity of illness and medication will affect mobility, physical status and cognition [10], requiring a special assessment in this setting in order to prevent falls. Moreover, an unknown environment like the hospital can contribute to increase previous risk or generate new risk factors.

The methodological weaknesses that have been identified are subsequently reflected in the under or over-detection of patients at risk of falling, and the routine use of such methods may divert attention and resources toward patients who would least benefit from preventive measures, at the expense of those who do need them.

These considerations highlight controversies and inconsistencies regarding the widespread use of these tools in the field of acute hospital care. Therefore, in order to dispel some of these uncertainties and to determine which of the available instruments offers better diagnostic performance for fall prevention as part of a range of preventive interventions to minimise risk among hospitalized patients, we present an up-to-date, detailed analysis of the existing literature illustrating the scope of measures available. The results of this review will contribute to the implementation of best practices related to preventing falls in an acute care hospital setting.

The aim of this review is to determine the accuracy of instruments for detecting fall risk and predicting falls in acute hospitalized patients. The specific objectives were to analyse the diagnostic validity and psychometric properties of the various risk assessment tools for predicting falls in acute hospitalized patients, and to compare the effectiveness of risk assessment instruments for falls and its impact on the incidence of falls by acute hospitalized patients.

Following the Cochrane Manual for Diagnostic Test Accuracy [29], this systematic review focuses on establishing the accuracy of instruments, scales or questionnaires (index) developed for detecting or predicting falls (target condition) in acute hospitalized patients, aged 16 or over (patients).

Thus, the review determines what instruments are available for assessing the risk of falls by acute hospitalized patients, the differences among them in terms of diagnostic accuracy and/or psychometric properties and their potential impact on preventing falls when implemented in the clinical context.

## Methods

### Study design

Systematic review, performed according to the recommendations of the Cochrane Handbook for Systematic Reviews of Interventions [29], and meta-analysis. This review focuses on three types of research papers: those which develop diagnostic validity (DV), those which accomplish psychometric validity (PV) and those which evaluate the effectiveness of fall risk assessment instruments (EFRA).

#### Inclusion/exclusion criteria

1.1  Types of studies

• For DV, diagnostic validation studies of falls risk assessment tools.

• For PV, observational studies that compare the validity and reliability of falls risk assessment tools.

• For EFRA, experimental studies, randomised or not, with a control group, including the use of a falls risk assessment tool and including comparison data for sensitivity, specificity, predictive values and/or likelihood ratios with respect to other instruments or professional clinical judgement (nurses, doctors, physiotherapists, etc.).

• Systematic reviews of either of these types of studies, if they meet the inclusion criteria for participants, interventions and outcomes.

1.2  Types of participants

For any of the three types of studies, only adult patients in acute hospitals are included:

• Adults (aged over 16 years) admitted to acute care hospitals.

• Studies focusing on patients admitted to acute psychiatric units or to paediatric units are excluded from this review.

Since this study focused on acute patients, patients living in the community, rehabilitation hospitals or rehabilitation units, sub-acute, long-stay patients, institutionalized patients, did not meet the inclusion criteria.

1.3  Types of intervention

In DV and PV studies the type of intervention criterion is not applicable. In EFRA studies, experimental studies involving the use of a falls risk assessment tool, either as a sole intervention or in conjunction with others, are accepted.

1.4  Types of outcome measure

In DV studies, any measure of diagnostic validity: sensitivity, specificity, predictive values, likelihood ratios, diagnostic Odds Ratio (DOR), area under the curve (AUC) and frequency and distribution of risk factors.

In PV studies, any psychometric outcome such as reliability, internal consistency, face, criterion or construct validity and frequency and distribution of risk factors.

In EFRA studies: frequency of falls during patients’ stay in hospital or falls predicted, complications resulting from falls, frequency and distribution of risk factors identified.

#### Search methods

The following databases were searched: MEDLINE, CINAHL, EMBASE, WEB OF SCIENCE, SCOPUS, COCHRANE, CRD, IME, CUIDEN PLUS, ENFISPO, LILACS, COCHRANE PLUS, together with these related websites: PRoFaNE (Prevention of Falls Network Europe), NSW Falls Prevention Network, Cochrane Bone, Joint and Muscle Trauma Group and Google Scholar. To avoid publication bias we also searched gray literature websites Open Grey, Teseo, Dart Europe and “Tesis Doctorales en Red” (TDR). The search languages were English, Spanish and Portuguese and the periods covered, from the date of the first study indexed in the corresponding database, up to and including 31 August 2011. In addition, linked searches were made in the references for the studies found. Search strategies are available as an additional file (see Additional file 1).

For the searches, we used specific methodological filters developed by the Health Information Research Unit at McMaster University for studies of diagnostic tools and clinical prediction rules [30,31]. Initially, the terms used were: accidental; falls; fallers; risk assessment; assessment tool; balance; gait; validation studies; prevention; prediction; hospital units; hospitals; acute care. In addition, we applied the terms needed to adjust the criteria for exclusion from the review, with the logical operator NOT (exclusion of studies in the community and those focusing on psychiatric, paediatric and other such institutions).

#### Review method

The first stage of our review included a detailed assessment of the titles and abstracts to determine whether each article met the requirements for inclusion. If there was any doubt, the full text of the article was assessed to decide whether it met these criteria. To ensure the quality of the process, all records were doubly evaluated, by two blinded reviewers.

After this initial process, all the references identified as potentially eligible were evaluated to see if they met the inclusion criteria for the review. This process was again carried out in parallel by two blinded reviewers. Any discrepancies that might arise in the process were resolved by discussion between the two evaluators, assisted by the intervention of a third expert, not otherwise involved in the project. Additionally, a pilot exercise was performed with the reviewers, for application of the inclusion criteria, on a sample of 15 items to reduce the risk of bias.

#### Quality appraisal

For PV studies the assessment was based on the quality criteria identified for health questionnaires [32]. These quality criteria addressed the content validity, internal consistency, criterion validity, construct validity, reproducibility, longitudinal validity, responsiveness, floor and ceiling effects and interpretability. For DV studies, the Critical Appraisal Skills Programme (CASP) for diagnostic studies was the tool selected [33]. For systematic reviews, the PRISMA standard was used [34,35].

#### Data abstraction

An electronic form was used to input the results of the studies included and evaluated, supported by the application RevMan 5.0.24 and included the following items: clinical characteristics and context of the study, participants (number, selection, age, sex, type of disease or condition), design, reference standard and target process, test and comparisons, monitoring and observations. In addition, and to obtain data for PV and EFRA studies, the following information was included: number of items comprising the assessment tool, number of subscales (if applicable), type of questions (dichotomous, Likert, semantic differences, etc.), cutoff points (if any), recommendations on training for use, recommended frequency of administration, time required for administration, reliability data, results from factorial analysis or concurrent validity. Also included on this form were the RevMan 5.0.24 check-list items for assessing the quality of diagnostic studies. Furthermore, for EFRA studies, data on intervention, randomisation, group allocation, follow-up and end-points were collected.

Prior agreement will be reached on possible codes to describe the standard outcome routines for these studies. When the original studies did not clearly provide the data necessary for analysis, the authors were contacted directly for clarification or for the exact data, if possible.

#### Synthesis

Meta-analyses of diagnostic Odds Ratios (DOR) and likelihood ratio (LH) coefficients were performed with the random effects method [36]. DOR combines positive and negative likelihood ratios and it represents a global performance measure: how greater is the odds of having the condition among those with a positive result with the instrument versus those ones with a negative result [37].

Forest plots were calculated for sensitivity and specificity, DOR and LH. Additionally, SROC (Summary Receiver Operating Characteristic) curves were calculated for every analysis through the square minimum weighted by the inverse of the variance. Heterogeneity among studies was addressed using forest-plot diagrams for sensitivity and specificity and the likelihood ratio test for these two dimensions. In addition, Cochrane’s Q statistic was calculated for the positive and negative probability ratios, using as weights the reciprocals of the variances and the I^2^ statistic. The latter value was calculated from the Q statistic (the standardised measure of the observed heterogeneity, which is not affected by effect size units). The heterogeneity was stratified into three levels, following the criteria of Higgins et al. [38]: <25% low heterogeneity, 25-50% moderate heterogeneity and >50% high heterogeneity.

To control the potential extra source of variability among studies resulting from potential differences among them regarding the thresholds for defining positive and negative results (threshold effect), we calculated Spearman’s correlation coefficient between sensitivity and specificity [39]. Prior to this, we determined whether or not the diagnostic odds ratio, using the Moses-Shapiro-Littemberg method to decide whether the points on a ROC curve should be adjusted symmetrically or asymmetrically, respectively [40]. As there was no threshold effect, the global sensitivity and specificity were calculated. Meta-regression models were developed introducing two co-variables: mean age over 65 and presence/absence of risk re-assessment along the admission period. This was carried out for exploring additional heterogeneity sources, by adding up co-variables to the model. The exponential transformation of the estimated coefficients can be interpreted as the relative DOR of that co-variable and it shows the change in the diagnostic performance when the co-variable varies [41].

A concordance analysis among reviewers was carried out during the different phases of the process and this was subsequently incorporated into the results of the review, using a Kappa index. For the different phases of analysis, the applications RevMan 5.0.24, MetaDiSc 1.1.1 and PASW 18 were used.

#### Ethical considerations

This study deals with secondary data from original studies and therefore is not subject to the usual criteria for original research. Nevertheless, the review participants signed an explicit statement that there is no conflict of interest.

## Results

**Figure 1:**
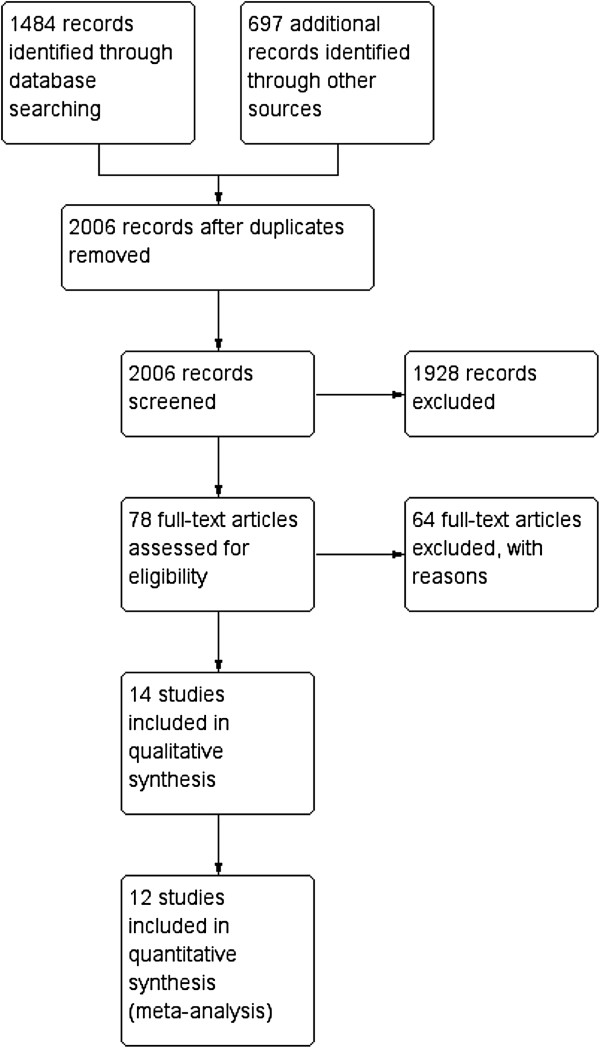
Study flow diagram.

A search within different databases and webs produced a total of 2,181 references (Table 1). After removing duplicates, there remained 2,006 articles, whose titles and abstracts were evaluated by blinded pairs of reviewers. After this first phase, 78 articles were selected as potentially eligible, and the full text was then read, again by blinded pairs, to assess its quality, extract data and determine its inclusion or otherwise in the meta-analysis. Finally, 14 studies [15,20,22,23,25-27,42-48] were selected for this review (Figure 1). A summary of the references and the reasons for excluding the remaining 64 items is provided in Table 2.

**Table 1:** Results of the bibliographic search

**Source**	**Articles located**
COCHRANE PLUS	28
DARE	41
SCOPUS	122
WEB OF SCIENCE	227
LILACS	71
ENFISPO	124
CUIDEN	119
EMBASE	107
CINAHL	250
PUBMED	288
Google Scholar	554
IME	97
ProFaNe	9
Cochrane bone	3
Cochrane Library	7
Linked searches	16
Dart Europe	1
TDR	39
Open grey	3
Teseo	75
**TOTAL**	**2181**

Number of references by databases or websites.

**Table 2:** Summary of the causes of exclusion of rejected studies

**REASON FOR EXCLUSION**					
**Does not meet inclusion criteria**	**This is not a validation study of an instrument for assessing the risk of falls**	**Provides no data or insufficient data to reproduce the calculations of diagnostic validity**	**It’s a comment from another article**	**The study fails the assessment of methodological quality**	**Language other than English, Spanish or Portuguese**
Brians 1991 [49]; Browne 2004 [50]; Chow 2007 [51]; Eagle 1999 [52]; El Miedany 2011 [53]; Gerdhem 2005 [54]; Haines 2006 [55]; Haines 2007 [56]; Harrington 2010 [57]; Heinze 2006 [58]; Heinze 2009 [59]; Hendrich 1995 [17]; Hendrich 2003 [18]; Hernández 2008 [60]; Hill 2004 [61]; Jester 2005 [62]; Lee 2011 [63]; Macavoy 1996 [64]; Mertens 2007 [65]; Mertens 2010 [66]; Morse 1988 [14]; Myers 2003 [24]; Myers&Nikoletti 2003 [67]; Nakagawa 2008 [68]; Oliver 2004 [9]; Oliver 2008 [69]; O’Connell 2002 [70]; Perell 2001 [10]; Petitpierre 2010 [71]; Price 1998 [72]; Roqueta 2007 [73]; Tew 2011 [74]; Toyabe 2010 [75]; Webster 2008 [76]; Webster 2010 [77]; Yauk 2005 [78];	Cina-Tschumi 2009 [79]; Currie 2004 [80]; Echevarría 2007 [81]; Forrester 1999 [82]; Giles 2006 [83]; Hendrich 1988 [84]; Hendrich 2007 [85]; Juvé 1999 [86]; Kinn 2001 [87]; McFarlane 2004 [88]; Parker 2000 [89]; Poe 2005 [90]; Webster&Courtney 2008 [91]; Wiens 2006 [92]	Agudelo 2010 [93]; Salameh 2008 [94]; Schwendimann 2006 B [95]	Agudelo 2009 [96]; Beghe 2007 [97]; Healey 2010 [98]; Kasseroler 2009 [99]	Conley 1999 [12]; McCollam 1995 [100]; Robeywilliams 2007 [101]	Caldara 2008 [102]; Chiari 2002 [103]; Días 2006 [104]; Salarvand 2010 [105]

References of excluded studies grouped by main reason for exclusion.

A total of 14,663 patients were considered in the studies selected, although several of the latter, in addition to the diagnostic validation of the instrument or instruments in question, also conducted studies of intra-observer reliability [48] inter-observer reliability [26,42,48] or the procedure leading to the development of some risk assessment scales [15,46]. A total of 13,284 patients were involved in the analysis of diagnostic accuracy. Although not all the studies provided the age and sex distribution of their populations, according to the published data these involved 5,504 men (41.43%) and 5,358 women (40.33%). All the studies were performed in hospitals for acutely-ill adults. Several of them focused on patients aged over 50 years [45], over 65 years [15,20,44], or on hospital departments that mainly treated the elderly [47]. In consequence, the overall mean age of the patients was 69.76 years (SD 9.56). In all cases, the diagnostic validation of the different risk assessment tools was performed prospectively. No experimental studies were conducted (Table 3).

**Table 3:** Characteristics of selected studies

**Study**	Participants (n = 13284) *	**Study design**	**Index and comparator test**	**Age**	**Men**	**Women**
				**Mean (SD) (years)**	**n(%)**	**n(%)**
TOTAL				69.76 (9.56) ^*^	5504 (41.43%)^*^	5358 (40.33%)^*^
Barker 2011 [42]	Phase I: 263 patients. Phase II 52 patients	Prospective cross-sectional study. Phase I: Assessment of predicitive accuracy; phase II: Assessment on inter-rater agreement.	The Northern Hospital Modified STRATIFY (TNH-STRATIFY) vs STRATIFY.	61.32 (20.65)	137 (52.09%)	126 (47.91%)
Chapman 2011 [43]	1540 patients.	Descriptive and comparative cross-sectional study.	The Maine Medical Center fall risk assessment, the New York-Presbiterian Fall and injury risk assessment tool, Morse Fall Scale and Hendrich II fall risk model.	n.a.	n.a.	n.a.
Ivziku 2011 [44]	179 patients.	Descriptive prospective study.	Hendrich Fall Risk Model II (HFRM II).	79.47 (9.5)	74 (41.34%)	105 (58.66%)
Kim EAN 2007 [26]	Validity study: 5489 patients. Reliability study: 144 patients	Prospective descriptive study.	Morse Fall Scale (MFS), St Thomas Risk Assessment Tool in Falling Elderly Inpatients (STRATIFY) and Hendrich II Fall Risk Model (HFRM II).	55 (19)	2842 (51.78%)	2647 (48.22%)
Kim KS 2011 [27]	356 patients.	Prospective cohort study.	Morse Fall Scale (MFS), Bobath Memorial Hospital Fall Risk Assessment Scale (BMFRAS), Johns Hopkins Hospital Fall Risk Assessment Tool (JHFRAT).	62.6 (n.a.)	201 (56.46%)	155 (43.54%)
Lovallo 2010 [45]	1148 patients.	Prospective observational study.	Conley Scale and Hendrich Fall Risk Model.	69 (10.33)	680 (59.23%)	468 (40.77%)
Milisen 2007 [23]	Total sample: 2568 patients; surgical wards: 875 patients; medical wards: 1006 patients.	Prospective multicenter study.	St. Thomas’s Risk Assessment Tool in Falling Elderly Inpatients (STRATIFY).	Medical wards: 64.1 (18); Surgical wards: 58.2 (17.1)	Medical wards: 494 (49.10%); Surgical wards: 439 (50.17%)	Medical wards: 512 (50.9%); Surgical wards: 436 (49.83%)
Oliver 1997 [15]	Phase 1: 116 cases and 116 controls; phase 2 (local validation): 217 patients; phase 3 (remote validation): 331 patients.	Phase 1: a prospective casecontrol study. Phases 2 and 3: prospective cohort study.	Development of STRATIFY.	n.a.	n.a.	n.a.
Papaioannou 2004 [20]	620 patients.	Prospective validation cohort study.	Weigthed STRATIFY vs Unweighted STRATIFY.	78 (7.7)	282 (45.48%)	338 (54.52%)
Schmid 1990 [46]	Phase 1: 204 patients; phase 2: 334 patients.	Phase 1: a retrospective casecontrol study. Phase 2: prospective cohort study.	Development of a new fall risk assessment tool.	n.a.	n.a.	n.a.
Schwendimann 2006 A [22]	386 patients.	Prospective cohort study.	Morse Fall Scale (MFS).	70.3 (18.5)	156 (40.41%)	230 (59.59%)
Schwendimann 2007 [25]	275 patients.	Prospective cohort study.	Morse Fall Scale (MFS).	80.3 (12.4)	99 (36%)	176 (64%)
Vassallo 2005 [47]	135 patients.	Prospective, open, observational study.	STRATIFY, Downton, Tullamore, and Tinetti.	83.8 (8.01)	49 (36.3%)	86 (63.7%)
Walsh 2010 [48]	130 inpatients in the predictive accuracy evaluation; 25 and 35 inpatients for the intra-rater and inter-rater reliability analyses.	Prospective cohort study of predictive validity and observational investigation of intra- and inter-rater reliability.	A new instrument (Western Health Falls Risk Assessment, WHeFRA) was compared with ‘gold standard tool’ (STRATIFY).	75 (29–94)^**^	51 (39.23%)	79 (60.77%)

Number of participants, study design, index and comparator test, mean age and gender of selected studies.

*Calculations with the available data.

**This study provided the age range but not SD.

Regarding the methodological quality of the studies, some shortcomings were identified, mainly related to two aspects: the lack of blinding in outcome assessment or lack of information in this respect, and doubt as to the representativeness of the study population, generally because the article failed to stipulate how the sample size was calculated (Table 4).

**Table 4:** Summary of the methodological evaluation of selected studies

	**Was there a comparison with an appropriate reference standard?**	**Was there an appropriate spectrum of patients?**	**Was there adequate description of the test?**	**Was there blind outcome assessment?**	**Decision to perform the gold standard, was independent of the test result?**	**Can likelihood ratios be calculated?**	**What was the accuracy of the results?**	**Can the results be applied to your patients?**	**Is the test acceptable in this case?**	**Will the results of the test change your actions?**
Barker 2011 [42]	**+**	**+**	**+**	**+**	**+**	**+**	**+**	**+**	**+**	**+**
Chapman 2011 [43]	**+**	**+**	**+**	**-**	**+**	**+**	**?**	**?**	**+**	**+**
Ivziku 2011 [44]	**+**	**?**	**+**	**-**	**+**	**+**	**+**	**+**	**+**	**+**
Kim EAN 2007 [26]	**+**	**+**	**+**	**+**	**+**	**+**	**+**	**+**	**+**	**+**
Kim KS 2011 [27]	**+**	**+**	**+**	**-**	**+**	**+**	**+**	**+**	**+**	**+**
Lovallo 2010 [45]	**+**	**+**	**+**	**-**	**+**	**+**	**+**	**+**	**+**	**+**
Milisen 2007 [23]	**+**	**+**	**+**	**?**	**+**	**+**	**+**	**+**	**+**	**+**
Oliver 1997 [15]	**+**	**+**	**+**	**+**	**+**	**+**	**+**	**+**	**+**	**+**
Papaioannou 2004 [20]	**+**	**+**	**+**	**+**	**+**	**+**	**+**	**+**	**+**	**+**
Schmid 1990 [47]	**+**	**?**	**+**	**?**	**+**	**+**	**?**	**+**	**+**	**+**
Schwendimann 2006A [22]	**+**	**?**	**+**	**-**	**+**	**+**	**+**	**+**	**+**	**+**
Schwendimann 2007 [25]	**+**	**?**	**+**	**?**	**+**	**+**	**+**	**+**	**+**	**+**
Vassallo 2005 [47]	**+**	**?**	**+**	**+**	**+**	**+**	**+**	**?**	**+**	**+**
Walsh 2010 [48]	**+**	**?**	**+**	**+**	**+**	**+**	**+**	**+**	**+**	**+**

“+”: positive evaluation; “-”: negative evaluation; “?”: no information about the item.

Although all the studies provided data enabling reproduction of the calculations of diagnostic validation, not all were included in the meta-analysis, because some scales did not contain sufficient studies for this (Figure 1). The Schmid study [46] was discarded because it described the development of an assessment tool for the risk of falls but this was not subsequently tested in any of the selected studies. We also excluded the Oliver study [15], which although it provided data on local validation and the remote validation of the STRATIFY scale, expressed the results in terms of ‘falls’ rather than ‘fallers’ as in all the selected studies. Therefore, and as done by this very author in a meta-analysis of the same scale [69], it was ruled out of our meta-analysis. With respect to the study by Milisen [23] we only considered the data for patients admitted to medical and surgical units (these data are available separately), but not to geriatric units, as the latter would not meet the criteria for inclusion in the present review, which is limited to acutely-ill patients.

**Figure 2:**
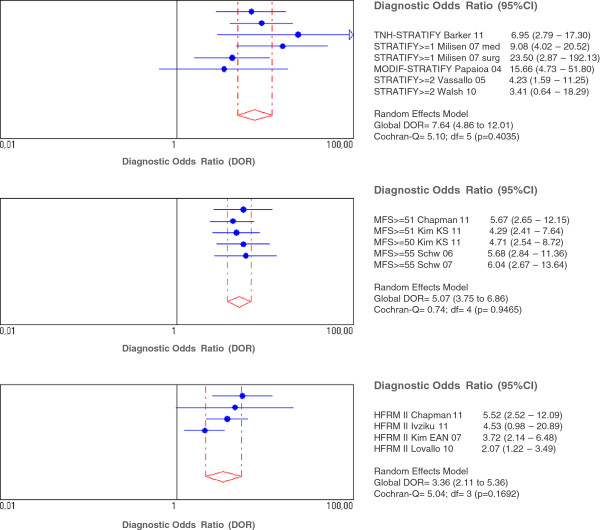
**Forest plots of diagnostic odds ratio of STRATIFY, MFS Y HFRM II tools.** *Forest plot were DOR of each individual study is represented by the blue point and its correspondent 95% confidence intervals. The rhombus and the scattered red lines represent the global DOR and its 95% CI, respectively.

Finally, the meta-analysis was performed with the Morse, STRATIFY and Hendrich II Fall Risk Model instruments. The results showed that the STRATIFY tool provided greater diagnostic validity, with a diagnostic odds ratio (DOR) value of 7.640 (95% CI: 4.862 - 12.007) versus 5.068 (95% CI: 3.747 - 6.857) for the MFS and 3.362 (95% CI: 2.107 - 5.364) for the HFRM II (Table 5). Figure 2 shows the forest plots with partial DOR of each study included into the meta-analysis, as well as the global DOR for each tool and the 95% confidence interval.

**Table 5:** Summary of the results of the meta-analysis

	**STRATIFY**	**MFS**	**Hendrich**
**Sensitivity (95% CI)**	0.800 (0.724 – 0.863)	0.755 (0.698 – 0.806)	0.628 (0.549 – 0.702)
**Specificity (95% CI)**	0.675 (0.658 – 0.692)	0.677 (0.659 – 0.695)	0.640 (0.630 – 0.651)
**LH + (95% CI)**	2.467 (2.047 – 2.973)	2.014 (1.800 – 2.254)	1.793 (1.500 – 2.142)
**LH- (95% CI)**	0.337 (0.224 – 0.507)	0.401 (0.324 – 0.498)	0.542 (0.367 – 0.802)
**DOR (95% CI)**	7.640 (4.862 – 12.007)	5.068 (3.747 – 6.857)	3.362 (2.107 – 5.364)

Results of sensitivity, specificity, LH+, LH- and DOR of the fall risk assessment tools with which conducted meta-analysis.

Sensitivity analyses performed for MFS showed that after removing the Kim EAN 2007 study [26], heterogeneity was markedly improved, possibly because it included younger patients (average age below 65 years), to whom lower cutoff points were applied (25 and 51). The same was true for the STRATIFY meta-analysis, where, as well as the Kim EAN 2007 study [26], the data referring to the original scale in the Barker 2011 study [42], were also removed. In both cases, the mean age of the study population was less than 65 years, although the cutoff points (2 and 3) were higher than those applied in the other study included in this meta-analysis with similar characteristics in terms of the age of the sample population, namely Milisen 2007 [23] (cutoff point, 1). The removal of these two studies significantly improved heterogeneity, although this remained high for specificity and for a positive likelihood ratio (LH+), without reaching statistical significance for the DOR. On the HFMR II tool, heterogeneity was over 50% in sensitivity and specificity, but was not significant for DOR, LH + or LH-.

A meta-regression was also performed, in order to assess the effect of average patient age over 65 years [20,22,25,44,45,47,48] and the performance or otherwise of risk reassessments during the patient’s stay, versus a single evaluation on admission [27,45]. In the case of the MFS, the reassessment showed a significant reduction in the DOR on the tool (rDOR 0.75; 95% CI: 0.64 - 0.89, p = 0.0176).

## Discussion

Although other systematic reviews and meta-analyses of assessment tools for falls by hospitalized patients have been performed [9,56,57,69,106], ours is the first that includes only acute patients. This is particularly useful in clinical practice for identifying the behaviour of the instruments currently used exclusively in the hospital environment, where falls are among the most frequent adverse events [5], and thus are directly relevant to the development and implementation of safety policies in acute care hospitals.

Since V. Scott [106] and T.P. Haines [56] published their systematic reviews of fall risk assessment tools in 2007, there were no new updates focused on that instruments concerning acute hospitalized patients. The systematic review published by Oliver in 2009 focused only on the STRATIFY tool and was not limited to acute patients. In the present review, 9 [23,25-27,42-45,48] of the 14 selected studies have been published since 2007, allowing an update of knowledge available on this topic. This is one of the strengths of this study. Another strength of this review is that contemplated studies assessing the psychometric properties of the fall risk assessment instruments.

This meta-analysis was carried out as a comparison of the Morse Fall Scale (MFS), the St. Thomas Risk Assessment Tool in Falling Elderly Inpatients (STRATIFY) and the Hendrich II Fall Risk Model (HFRM II). The results obtained showed the STRATIFY tool to be the best tool for assessing the risk of falls among hospitalized acutely ill adult patients, followed by MFS and finally HFRM II. STRATIFY produced the best values for sensitivity and had a specificity similar to that of MFS, and obtained the best values for DOR. In part, these results contradict those published in a recent meta-analysis focusing on MFS and STRATIFY [57], which found a higher sensitivity but lower specificity for the MFS with respect to STRATIFY. However, these results did not include the calculation of the DOR and some of the studies that were included were excluded from our analysis as not meeting the inclusion criteria. Moreover, we also considered another four studies published subsequently with data for these assessment tools [27,42,43,48].

These three tools have been compared by their simultaneous application in a sample of hospitalized adult patients [26]. In this study, HFRM II was found to be the most suitable for identifying the patients at high risk of falls, with a sensitivity of 70% and a specificity of 61.8%. However, in the present meta-analysis, HFRM II proved to be the worst of the three instruments considered, due to its lower sensitivity (0.628), specificity (0.640) and DOR (3.362).

In another study in which four risk assessment instruments (STRATIFY, Tinetti, Downton and Tullamore) were tested simultaneously in an acute patient hospital environment [47], STRATIFY was completed most easily and in the least time; furthermore, it also presented the best predictive validity, although it was the least sensitive of the four. The short time required to administer this assessment scale and the fact that it is readily understandable for medical staff are very relevant factors in an acute hospital setting where work loads are high and periodic reassessments of patients are advisable.

In previous systematic reviews of this question, one of the inclusion criteria was that the selected studies should conduct a prospective validation of assessment tools for falls [9,69,106]. In the systematic review and meta-analysis carried out by Haines in 2007 [56] one of the practical implications described was that although retrospective evaluations are still valuable for generating initial results and identifying the tools and cutoff points that may be useful in clinical practice, less weight should be given to their results than to those obtained from prospective studies, with respect to selecting a detection tool for use in clinical practice. In coherence with this view, all the studies included in the present review conducted a prospective validation of the various instruments examined. Nine of the fourteen studies had been published since the completion of the above-mentioned systematic review. Moreover, the present analysis complies with one of the “gold standard” criteria described by Wyatt and Altman for such scales [107], although in none of the articles selected was a randomised controlled trial carried out, and this may be an area for improvement regarding the development of future research in this field.

Previous studies have argued that fall risk assessment performed only at the time of hospital admission does not identify changes in the patients’ clinical status during hospitalisation, although this is a common occurrence, especially among the elderly, who may become disoriented, agitated or lose functionality during hospitalisation, and thus be at greater risk of suffering a fall [12]. The acute phase of the disease and changes in medication can affect both mobility and the physical and cognitive status, and therefore hospitals need an instrument that can be used quickly and easily so that repeated assessments of these patients may be carried out [10]. In only four of the fourteen studies selected for this review was a reassessment conducted, whether on a weekly schedule [15,27,46] or following changes in the patient’s condition, after cognitive impairment, after significant changes in medication or after a fall [45]. Although the meta-regression analysis with respect to this criterion showed no effect for HFRM II, and could not be performed for STRATIFY due to the lack of studies in which a reassessment of patients was performed, in the case of MFS, the reassessment produced a significant reduction in the DOR. This may be explained, in part, as the MFS losing predictive capacity when the risk of patients’ suffering a fall decreases, as their condition improves. In the only study in which a reassessment was carried out with MFS [27] the mean age of the patients was below 65 years (62.6). The condition of these younger patients would presumably improve over time, and so their risk is more difficult to identify with this scale. However, as discussed above, in only one of the studies in which MFS was tested was a reassessment performed. Thus, further research is needed, including reassessment both with MFS and with the other instruments in order to achieve a more realistic analysis of this circumstance. It should be borne in mind that, in general, it is difficult to accurately predict the risk of falls among hospitalized adult patients who are subjected to external risk factors, specific to the hospital environment and which are not taken into account by any of the assessment instruments described.

This review and meta-analysis may also be affected by the limitations of the primary studies analysed. First, knowledge of the number of patients suffering a fall is always dependent on the voluntary reporting of this fact by the healthcare staff, and so falls may occur that are not reported, which would to some extent invalidate the results obtained in these studies. Second, the review may be affected by contamination related to the implementation of other actions taken to prevent falls in the different environments studied, and by a possible Hawthorne effect. Moreover, limitations arise from the questionable quality of some of the studies selected: some offered no data on the age and/or sex distribution of the study population [15,43,46], or were deficient regarding the representativeness of the sample [22,25,44,46-48] or regarding the blinding of the researchers [22,27,43-45]. Another possible limitation concerns the search language: in the present review, the search languages used were limited to English, Spanish and Portuguese, and four studies were excluded for this reason [102-105].

In short, despite the findings obtained, our analysis of the various studies clearly shows that the behaviour of these risk assessment instruments varies considerably depending on the population and the environment in which they are administered. In consequence, we cannot recommend the generalised adoption of any single method without its prior testing in the healthcare setting of the intended implementation. Moreover, it should be recalled that these instruments, or the actions taken including their use, will not be effective if healthcare personnel do not ensure patient safety procedures are followed, and this aspect remains to be investigated in the case of falls by hospitalized patients. A study of safety and security in Spanish hospitals reported that the majority of healthcare staff (77.8%) had not reported any event related to patient safety in the past year and that 95% had reported fewer than two such events [108]. This aspect, noted above as one of the limitations of our study, and the question of compliance by personnel with procedures established to prevent adverse events, are issues which must be addressed in order to achieve an effective culture of safety within hospitals.

## Conclusions

The STRATIFY scale was found to be the best tool for assessing the risk of falls by hospitalized acutely-ill adults. With this scale, the DOR was higher than with the MFS and HFRM II. However, the behaviour of these instruments varies considerably depending on the population and the environment, and so their operation should be tested prior to implementation. Further studies are needed to investigate the effect of the reassessment of these instruments with respect to hospitalized adult patients, and to consider the real compliance by healthcare personnel with procedures related to patient safety, and in particular concerning the prevention of falls.

## Abbreviations

AUC: Area under the curve; CASP: Critical appraisal skills programme; CI: Confidence interval; DOR: Diagnostic odds ratio; DV: Diagnostic validity; EFRA: Effectiveness of fall risk assessment instruments; HFRM: Hendrich fall risk model; JCI: Joint commission international; LH: Likelihood ratio; MFS: Morse fall scale; PRISMA: Preferred reporting items for systematic reviews and meta-analyses; PV: Psychometric validity; rDOR: Reduction in diagnostic odds ratio; RevMan: Review Manager; SD: Standard deviation; SROC: Summary receiver operating characteristic; STRATIFY: St. Thomas risk assessment tool in falling elderly inpatients.

## Competing interests

The authors declare that they have no competing interests.

## Authors’ contributions

JMMA and MAG designed the study. MAG, JCCS, SBS, CPJ, AMF, MELR, ABMS and AMMB were involved in data collection and evaluation. JMMA performed the statistical analysis. All authors helped with the interpretation of the data. MAG and JMMA drafted the first version of the manuscript, and all authors contributed to subsequent versions and revised it critically for important intellectual content. All authors read and approved the final manuscript.

## Pre-publication history

The pre-publication history for this paper can be accessed here:

http://www.biomedcentral.com/1472-6963/13/122/prepub

## Supplementary Material

Additional file 1 **Search strategies.** Search strategies on databases and webs, with number of references obtained in each of them. Click here for file
