# Supplementary material for: Instruments for assessing the risk of falls in acute hospitalized patients: a systematic review and meta-analysis
Source: BMC Health Serv Res. 2013 Apr 2;13:122. doi: 10.1186/1472-6963-13-122 (PMC3637640; doi:10.1186/1472-6963-13-122)
Supplement: Additional file 1 — Search strategies. Search strategies on databases and webs, with number of references obtained in each of them. [file 1472-6963-13-122-S1.docx]

**SEARCH STRATEGIES:**

**MEDLINE (from PubMed):** Search on May 26, 2011; 288 references.

#1 Search ((((((((((("Inpatients"[Majr])) AND ((((((((((((((((((((((((((((((((("falls"[Title/Abstract]) OR "falls/accidents"[Title/Abstract]) OR "falls/admission"[Title/Abstract]) OR "falls/fallers"[Title/Abstract]) OR "falls/mobility"[Title/Abstract]) OR "falls/patient"[Title/Abstract]) OR "falls/prevention"[Title/Abstract]) OR "falls/recurrent"[Title/Abstract]) OR "falls accidents"[Title/Abstract]) OR "falls assessment"[Title/Abstract]) OR "falls efficacy scale"[Title/Abstract]) OR "falls frequency"[Title/Abstract]) OR "falls incidence"[Title/Abstract]) OR "falls injury prevention"[Title/Abstract]) OR "falls intervention"[Title/Abstract]) OR "falls monitoring"[Title/Abstract]) OR "falls prediction"[Title/Abstract]) OR "falls prevention"[Title/Abstract]) OR "falls prevention activities"[Title/Abstract]) OR "falls prevention activity"[Title/Abstract]) OR "falls prevention intervention"[Title/Abstract]) OR "falls prevention interventions"[Title/Abstract]) OR "falls prevention research"[Title/Abstract]) OR "falls prevention strategies survey"[Title/Abstract]) OR "falls rate"[Title/Abstract]) OR "falls rates"[Title/Abstract]) OR "falls relative risk"[Title/Abstract]) OR "falls risk"[Title/Abstract]) OR "falls risk assessment"[Title/Abstract]) OR "falls risk group"[Title/Abstract]) AND (Humans[Mesh] AND adult[MeSH]))) OR ("Accidental Falls"[Majr] AND (Humans[Mesh] AND adult[MeSH]))))) OR ((((("Inpatients"[Majr])) AND ((((((((((((((((((((((((((((((((("falls"[Title/Abstract]) OR "falls/accidents"[Title/Abstract]) OR "falls/admission"[Title/Abstract]) OR "falls/fallers"[Title/Abstract]) OR "falls/mobility"[Title/Abstract]) OR "falls/patient"[Title/Abstract]) OR "falls/prevention"[Title/Abstract]) OR "falls/recurrent"[Title/Abstract]) OR "falls accidents"[Title/Abstract]) OR "falls assessment"[Title/Abstract]) OR "falls efficacy scale"[Title/Abstract]) OR "falls frequency"[Title/Abstract]) OR "falls incidence"[Title/Abstract]) OR "falls injury prevention"[Title/Abstract]) OR "falls intervention"[Title/Abstract]) OR "falls monitoring"[Title/Abstract]) OR "falls prediction"[Title/Abstract]) OR "falls prevention"[Title/Abstract]) OR "falls prevention activities"[Title/Abstract]) OR "falls prevention activity"[Title/Abstract]) OR "falls prevention intervention"[Title/Abstract]) OR "falls prevention interventions"[Title/Abstract]) OR "falls prevention research"[Title/Abstract]) OR "falls prevention strategies survey"[Title/Abstract]) OR "falls rate"[Title/Abstract]) OR "falls rates"[Title/Abstract]) OR "falls relative risk"[Title/Abstract]) OR "falls risk"[Title/Abstract]) OR "falls risk assessment"[Title/Abstract]) OR "falls risk group"[Title/Abstract]) AND (Humans[Mesh] AND adult[MeSH]))) OR ("Accidental Falls"[Majr] AND (Humans[Mesh] AND adult[MeSH]))))) AND ("Risk Assessment"[Majr])))) OR (fall* AND Risk* AND Assessment* AND hospital* AND inpatient*)) OR ("Accidental Falls"[Majr] AND "Risk Assessment"[Majr])) OR ((((("Inpatients"[Majr])) AND ("Hospitals"[Majr]))) AND ("Accidental Falls"[Majr] AND "Risk Assessment"[Majr]))) OR ((("Hospitals"[Majr])) AND ("Accidental Falls"[Majr] AND "Risk Assessment"[Majr]))) NOT PSYCH*) NOT (PEDIAT* OR PAEDIAT*) 289

#2 Search ((instrumentation[sh] OR methods[sh] OR Validation Studies[pt] OR Comparative Study[pt] OR “psychometrics”[MeSH] OR psychometr*[tiab] OR clinimetr*[tw] OR clinometr*[tw] OR “outcome assessment (health care)”[MeSH] OR outcome assessment[tiab] OR outcome measure*[tw] OR “observer variation”[MeSH] OR observer variation[tiab] OR “Health Status Indicators”[Mesh] OR “reproducibility of results”[MeSH] OR reproducib*[tiab] OR “discriminant analysis”[MeSH] OR reliab*[tiab] OR unreliab*[tiab] OR valid*[tiab] OR coefficient[tiab] OR homogeneity[tiab] OR homogeneous[tiab] OR “internal consistency”[tiab] OR (cronbach*[tiab] AND (alpha[tiab] OR alphas[tiab])) OR (item[tiab] AND (correlation*[tiab] OR selection*[tiab] OR reduction*[tiab])) OR agreement[tiab] OR precision[tiab] OR imprecision[tiab] OR “precise values”[tiab] OR test–retest[tiab] OR (test[tiab] AND retest[tiab]) OR (reliab*[tiab] AND (test[tiab] OR retest[tiab])) OR stability[tiab] OR interrater[tiab] OR inter-rater[tiab] OR intrarater[tiab] OR intra-rater[tiab] OR intertester[tiab] OR inter-tester[tiab] OR intratester[tiab] OR intra-tester[tiab] OR interobserver[tiab] OR inter-observer[tiab] OR intraobserver[tiab] OR intra-observer[tiab] OR intertechnician[tiab] OR inter-technician[tiab] OR intratechnician[tiab] OR intra-technician[tiab] OR interexaminer[tiab] OR inter-examiner[tiab] OR intraexaminer[tiab] OR intra-examiner[tiab] OR interassay[tiab] OR inter-assay[tiab] OR intraassay[tiab] OR intra-assay[tiab] OR interindividual[tiab] OR inter-individual[tiab] OR intraindividual[tiab] OR intra-individual[tiab] OR interparticipant[tiab] OR inter-participant[tiab] OR intraparticipant[tiab] OR intra-participant[tiab] OR kappa[tiab] OR kappa’s[tiab] OR kappas[tiab] OR repeatab*[tiab] OR ((replicab*[tiab] OR repeated[tiab]) AND (measure[tiab] OR measures[tiab] OR findings[tiab] OR result[tiab] OR results[tiab] OR test[tiab] OR tests[tiab])) OR generaliza*[tiab] OR generalisa*[tiab] OR concordance[tiab] OR (intraclass[tiab] AND correlation*[tiab]) OR discriminative[tiab] OR “known group”[tiab] OR factor analysis[tiab] OR factor analyses[tiab] OR dimension*[tiab] OR subscale*[tiab] OR (multitrait[tiab] AND scaling[tiab] AND (analysis[tiab] OR analyses[tiab])) OR item discriminant[tiab] OR interscale correlation*[tiab] OR error[tiab] OR errors[tiab] OR “individual variability”[tiab] OR (variability[tiab] AND (analysis[tiab] OR values[tiab])) OR (uncertainty[tiab] AND (measurement[tiab] OR measuring[tiab])) OR “standard error of measurement”[tiab] OR sensitiv*[tiab] OR responsive*[tiab] OR ((minimal[tiab] OR minimally[tiab] OR clinical[tiab] OR clinically[tiab]) AND (important[tiab] OR significant[tiab] OR detectable[tiab]) AND (change[tiab] OR difference[tiab])) OR (small*[tiab] AND (real[tiab] OR detectable[tiab]) AND (change[tiab] OR difference[tiab])) OR meaningful change[tiab] OR “ceiling effect”[tiab] OR “floor effect”[tiab] OR “Item response model”[tiab] OR IRT[tiab] OR Rasch[tiab] OR “Differential item functioning”[tiab] OR DIF[tiab] OR “computer adaptive testing”[tiab] OR “item bank”[tiab] OR “cross-cultural equivalence”[tiab])) AND "Accidental Falls"[Mesh] 4596

#3 Search (instrumentation[sh] OR methods[sh] OR Validation Studies[pt] OR Comparative Study[pt] OR “psychometrics”[MeSH] OR psychometr*[tiab] OR clinimetr*[tw] OR clinometr*[tw] OR “outcome assessment (health care)”[MeSH] OR outcome assessment[tiab] OR outcome measure*[tw] OR “observer variation”[MeSH] OR observer variation[tiab] OR “Health Status Indicators”[Mesh] OR “reproducibility of results”[MeSH] OR reproducib*[tiab] OR “discriminant analysis”[MeSH] OR reliab*[tiab] OR unreliab*[tiab] OR valid*[tiab] OR coefficient[tiab] OR homogeneity[tiab] OR homogeneous[tiab] OR “internal consistency”[tiab] OR (cronbach*[tiab] AND (alpha[tiab] OR alphas[tiab])) OR (item[tiab] AND (correlation*[tiab] OR selection*[tiab] OR reduction*[tiab])) OR agreement[tiab] OR precision[tiab] OR imprecision[tiab] OR “precise values”[tiab] OR test–retest[tiab] OR (test[tiab] AND retest[tiab]) OR (reliab*[tiab] AND (test[tiab] OR retest[tiab])) OR stability[tiab] OR interrater[tiab] OR inter-rater[tiab] OR intrarater[tiab] OR intra-rater[tiab] OR intertester[tiab] OR inter-tester[tiab] OR intratester[tiab] OR intra-tester[tiab] OR interobserver[tiab] OR inter-observer[tiab] OR intraobserver[tiab] OR intra-observer[tiab] OR intertechnician[tiab] OR inter-technician[tiab] OR intratechnician[tiab] OR intra-technician[tiab] OR interexaminer[tiab] OR inter-examiner[tiab] OR intraexaminer[tiab] OR intra-examiner[tiab] OR interassay[tiab] OR inter-assay[tiab] OR intraassay[tiab] OR intra-assay[tiab] OR interindividual[tiab] OR inter-individual[tiab] OR intraindividual[tiab] OR intra-individual[tiab] OR interparticipant[tiab] OR inter-participant[tiab] OR intraparticipant[tiab] OR intra-participant[tiab] OR kappa[tiab] OR kappa’s[tiab] OR kappas[tiab] OR repeatab*[tiab] OR ((replicab*[tiab] OR repeated[tiab]) AND (measure[tiab] OR measures[tiab] OR findings[tiab] OR result[tiab] OR results[tiab] OR test[tiab] OR tests[tiab])) OR generaliza*[tiab] OR generalisa*[tiab] OR concordance[tiab] OR (intraclass[tiab] AND correlation*[tiab]) OR discriminative[tiab] OR “known group”[tiab] OR factor analysis[tiab] OR factor analyses[tiab] OR dimension*[tiab] OR subscale*[tiab] OR (multitrait[tiab] AND scaling[tiab] AND (analysis[tiab] OR analyses[tiab])) OR item discriminant[tiab] OR interscale correlation*[tiab] OR error[tiab] OR errors[tiab] OR “individual variability”[tiab] OR (variability[tiab] AND (analysis[tiab] OR values[tiab])) OR (uncertainty[tiab] AND (measurement[tiab] OR measuring[tiab])) OR “standard error of measurement”[tiab] OR sensitiv*[tiab] OR responsive*[tiab] OR ((minimal[tiab] OR minimally[tiab] OR clinical[tiab] OR clinically[tiab]) AND (important[tiab] OR significant[tiab] OR detectable[tiab]) AND (change[tiab] OR difference[tiab])) OR (small*[tiab] AND (real[tiab] OR detectable[tiab]) AND (change[tiab] OR difference[tiab])) OR meaningful change[tiab] OR “ceiling effect”[tiab] OR “floor effect”[tiab] OR “Item response model”[tiab] OR IRT[tiab] OR Rasch[tiab] OR “Differential item functioning”[tiab] OR DIF[tiab] OR “computer adaptive testing”[tiab] OR “item bank”[tiab] OR “cross-cultural equivalence”[tiab]) 5645395

# 4 Search (#2) AND #3 4596

#5 Search (#2) OR #3 5645395

#6 Search accidental falls[MeSH Major Topic] 6327

#7 Search (#6) AND #5 2543

#8 Search "Inpatients"[Majr] 4517

#9 Search (#7) AND #8 55

#10 Search "Hospitals"[Majr] 92668

#11 Search (#7) AND #10 38

#12 Search #7 Limits: All Adult: 19+ years 2102

#13 Search (#10) OR #8 Limits: All Adult: 19+ years 15621

#14 Search #13 NOT( (PEDIAT* OR PAEDIAT*) OR (PSYCH*)) Limits: All Adult: 19+ years 11624

#15 Search (#14) AND #12 Limits: All Adult: 19+ years 51

#16 Search (#15) OR #1 Limits: All Adult: 19+ years 264

#17 Search (((("Inpatients"[Majr])) AND ((((((((((((((((((((((((((((((((("falls"[Title/Abstract]) OR "falls/accidents"[Title/Abstract]) OR "falls/admission"[Title/Abstract]) OR "falls/fallers"[Title/Abstract]) OR "falls/mobility"[Title/Abstract]) OR "falls/patient"[Title/Abstract]) OR "falls/prevention"[Title/Abstract]) OR "falls/recurrent"[Title/Abstract]) OR "falls accidents"[Title/Abstract]) OR "falls assessment"[Title/Abstract]) OR "falls efficacy scale"[Title/Abstract]) OR "falls frequency"[Title/Abstract]) OR "falls incidence"[Title/Abstract]) OR "falls injury prevention"[Title/Abstract]) OR "falls intervention"[Title/Abstract]) OR "falls monitoring"[Title/Abstract]) OR "falls prediction"[Title/Abstract]) OR "falls prevention"[Title/Abstract]) OR "falls prevention activities"[Title/Abstract]) OR "falls prevention activity"[Title/Abstract]) OR "falls prevention intervention"[Title/Abstract]) OR "falls prevention interventions"[Title/Abstract]) OR "falls prevention research"[Title/Abstract]) OR "falls prevention strategies survey"[Title/Abstract]) OR "falls rate"[Title/Abstract]) OR "falls rates"[Title/Abstract]) OR "falls relative risk"[Title/Abstract]) OR "falls risk"[Title/Abstract]) OR "falls risk assessment"[Title/Abstract]) OR "falls risk group"[Title/Abstract]) AND (Humans[Mesh] AND adult[MeSH]))) OR ("Accidental Falls"[Majr] AND (Humans[Mesh] AND adult[MeSH]))))) OR ((((("Inpatients"[Majr])) AND ((((((((((((((((((((((((((((((((("falls"[Title/Abstract]) OR "falls/accidents"[Title/Abstract]) OR "falls/admission"[Title/Abstract]) OR "falls/fallers"[Title/Abstract]) OR "falls/mobility"[Title/Abstract]) OR "falls/patient"[Title/Abstract]) OR "falls/prevention"[Title/Abstract]) OR "falls/recurrent"[Title/Abstract]) OR "falls accidents"[Title/Abstract]) OR "falls assessment"[Title/Abstract]) OR "falls efficacy scale"[Title/Abstract]) OR "falls frequency"[Title/Abstract]) OR "falls incidence"[Title/Abstract]) OR "falls injury prevention"[Title/Abstract]) OR "falls intervention"[Title/Abstract]) OR "falls monitoring"[Title/Abstract]) OR "falls prediction"[Title/Abstract]) OR "falls prevention"[Title/Abstract]) OR "falls prevention activities"[Title/Abstract]) OR "falls prevention activity"[Title/Abstract]) OR "falls prevention intervention"[Title/Abstract]) OR "falls prevention interventions"[Title/Abstract]) OR "falls prevention research"[Title/Abstract]) OR "falls prevention strategies survey"[Title/Abstract]) OR "falls rate"[Title/Abstract]) OR "falls rates"[Title/Abstract]) OR "falls relative risk"[Title/Abstract]) OR "falls risk"[Title/Abstract]) OR "falls risk assessment"[Title/Abstract]) OR "falls risk group"[Title/Abstract]) AND (Humans[Mesh] AND adult[MeSH]))) OR ("Accidental Falls"[Majr] AND (Humans[Mesh] AND adult[MeSH]))))) AND ("Risk Assessment"[Majr])) 114

#18 Search (#16) AND #17 104

#19 Search (#16) OR #17 288

**CINHAL:** search on May 25, 2011; 250 references.

S1 (MM "Accidental Falls") (5181)

S2 (MM "Fall Risk Assessment Tool") (10)

S3 (MM "Hendrich Fall Risk Model") (4)

S4 (MM "Morse Fall Scale") (3)

S5 (MM "Safety Behavior: Fall Prevention (Iowa NOC)") (1)

S6 psych* (272906)

S7 (paedia* OR pedia*) (51398)

S8 (MM "Hospital Units") (1360)

S9 hospital OR s8 (117065)

S10 (S1 or S2 or S3 or S4 or S5) AND s9 (732)

S11 s7 OR s6 (316821)

S12 s10 not s11 (628)

S13 s10 not s11

Especificar por SubjectAge3: - Adult: 19-44 years

Especificar por SubjectAge2: - Middle Aged: 45-64 years

Especificar por SubjectAge1: - Aged, 80 and over

Especificar por SubjectAge0: - Aged: 65+ years (250)

**EMBASE:** search on May 24, 2011; 107 references.

#1 ‘falling’/mj AND ‘risk assessment’/exp/mj AND ‘hospital’/mj 3

#2 ‘fall risk assessment’/mj AND ‘hospital’/mj 0

#3 ‘fall risk assessment’/mj 147

#4 #1 OR #3 150

#5 ‘hospital patient’/mj 6326

#6 #4 AND #5 1

#7 #1 OR #3 AND ([adolescent]/lim OR [adult]/lim OR [aged]/lim)

AND [humans]/lim AND [embase]/lim 107

**COCHRANE PLUS:** search on June 9, 2011; 28 references.

#1 FALL*:TA 8142

#2 ((ADVERSE EVENT) OR (RISK ASSESSMENT)):TA 7004

#3 #1 AND #2 157

#4 HOSPITAL*:TA 44687

#5 #3 AND #4 36

#6 COMMUNITY:TA 10792

#7 #5 NOT #6 28

**ENFISPO:** search on June 27, 2011**;** 124 references.

Caída$ (searched in any field)

**CUIDEN PLUS:** search on July 5, 2011; 119 references.

caída AND hospital (title and abstract)

**SCOPUS:** search on July 7, 2011; 122 references.

((TITLE-ABS-KEY(assessment tool)) AND ((TITLE-ABS-KEY(risk) AND TITLE-ABS-KEY(fall*) AND TITLE-ABS-KEY(hospital*)))) AND NOT (TITLE-ABS-KEY((community OR psych* OR pediatric* OR child*)))

**CRD/DARE:** search on July 7**,** 2011; 41 references.

1 (risk) OR (fall*) OR (hospital*) IN DARE 9959

2 (risk) AND (fall*) AND (hospital*) IN DARE 70

3 (risk) AND (fall*) AND (hospital*) AND (prevention) IN DARE 32

4 (assessment) AND (tool) IN DARE 915

5 #2 AND #4 7

6 #3 AND #4 3

7 #5 OR #6 7

8 #2 OR #7 70

9 MeSH DESCRIPTOR undefined EXPLODE ALL TREES 0

10 MeSH DESCRIPTOR Accidental Falls WITH QUALIFIER PC IN DARE 46

11 #8 OR #10 100

12 MeSH DESCRIPTOR Hospital Units EXPLODE ALL TREES IN DARE 105

13 MeSH DESCRIPTOR Hospitalization EXPLODE ALL TREES IN DARE 439

14 MeSH DESCRIPTOR Inpatients IN DARE 38

15 #10 AND #14 1

16 #3 OR #15 33

17 MeSH DESCRIPTOR Risk Assessment EXPLODE 1 IN DARE 0

18 MeSH DESCRIPTOR Risk Assessment IN DARE 555

19 #16 AND #18 5

20 #16 OR #19 33

21 MeSH DESCRIPTOR Risk Factors IN DARE 977

22 #10 AND #14 AND #21 0

23 #10 AND #21 13

24 #20 OR #23 41

25 #16 OR #24 41

**WEB OF SCIENCE:** search on August 2, 2011; 227 references.

# 1: Topic=(risk fall): 19.641

# 2: TS=ASSESSMENT: 692.630

# 3: #2 AND #1: 2.842

# 4: TS=HOSPITAL: 415.004

# 5: #4 AND #3: 393

# 6: TS=CHILDREN: 868.847

# 7: #5 NOT #6: 368

# 8: TS=PSYCHIATR*: 161.691

# 9: #7 NOT #8: 357

# 10: TS=COMMUNITY: 506.628

# 11: #9 NOT #10: 229

# 12: TI=(risk fall assessment hospital): 10

# 13: #12 OR #11: 230

# 14: TI=(PREVENTION RISK FALL): 57

# 15: #14 OR #13: 285

# 16: TI=(prediction fall risk assessment): 1

# 17: #16 OR #15: 285

# 18: TI=(prevention assessment tool fall risk): 0

# 19: TI=(prevention assessment tool fall risk): 0

# 20: TI=( assessment fall risk): 135

# 21: #20 OR #17: 391

# 22: TI=(children OR community OR PSYCHIATR*): 717.153

# 23: #22 NOT #21: 717.131

# 24: #21 NOT #22: 369

# 25: #24 OR #5: 524

# 26: #20 OR #16 OR #14 OR #12: 185

# 27: #26 NOT #22: 163

# 28: #27 OR #11: 369

# 29: TS=PATIENTS: 3.051.632

# 30: TI=PATIENT*: 1.225.570

# 31: #30 OR #29: 3.052.318

# 32: #31 AND #28: 225

# 33: TI=(SCAL* OR TOOLS*): 277.507

# 34: #33 AND #32: 9

# 35 : #34 OR #32: 227

**IME**: search on July 29, 2011; 97 references.

Parameters: Abstract=''escala*'' OR Abstract=''riesgo*'' Y Abstract=''caída*''

**GOOGLE ACADÉMICO:** search on July 19, 2011; 554 references.

caidas AND eventos adversos AND seguridad clínica AND valoración riesgos AND paciente hospitalizado AND estudio validación

**LILACS:** search on August 2, 2011; 71 references.

*SEARCH IN SPANISH: 54 REFERENCES*

"RIESGO" or "RIESGO-PREVENCION" or "RIESGO." or "RIESGO.." or "RIESGO/" or "RIESGO/PREDICTORES" or "RIESGOASOCIADAS" or "RIESGOASOCIADOS" or "RIESGODE" or "RIESGOS" or "RIESGOS.." or "RIESGOS/" [Palabras] and ( ( ( caídas ) or "CAIDA" ) or "CAIDA/" ) or "CAIDAS" or "CAIDAS.." or "CAIDAS/" [Palabras] and ( ( hospital ) or "HOSPITAL" ) or "HOSPITALES" [Palabras]

*SEARCH IN ENGLISH: 17 REFERENCES*

"RISK" or "RISK ASSESSMENT" or "RISK ASSESSMENT/" or "RISK FACTORS" or "RISK FACTORS/" or "RISK INDEX" or "RISK INDEX/" or "RISK MANAGEMENT" or "RISK MANAGEMENT/" or "RISK RATE" or "RISK RATE/" or "RISK RATIO" or "RISK RATIO/" or "RISK REDUCTION" or "RISK’." or "RISK’S" or "RISK-ASSESSMENT" or "RISK-BENEFIT" or "RISK-FACTOR" or "RISK-FACTORS" or "RISK-PREDICTING" or "RISK-PREDICTION" or "RISK-PROCTETION" or "RISK-RATIO" or "RISK-REDUCING" or "RISK-REDUCTION" or "RISK-RELATED" or "RISK." or "RISK.." or "RISK/" or "RISK/PROTECTION" or "RISKFACTOR" or "RISKFACTORS" [Words] and "FALL" or "FALL/" or "FALLED" or "FALLEN" or "FALLER" or "FALLERS" or "FALLING" or "FALLING.." or "FALLINGS" [Words] and "HOSPITAL" or "HOSPITAL UNITS" or "HOSPITAL UNITS/" or "HOSPITAL’" or "HOSPITAL’" or "HOSPITAL’." or "HOSPITAL’S" or "HOSPITAL-PACIENTE" or "HOSPITAL-PACIENTE/" or "HOSPITAL-PATIENT" or "HOSPITAL." or "HOSPITAL.." or "HOSPITAL/" [Words]

**Tesis Doctorales en Red (TDR):** search on June 1, 2011; 39 references.

(riesgo caídas) AND (hospital)

**DART Europe: E-theses portal:** search on June 1, 2011; 1 reference.

Fall* risk assessment

(Every country, every university and every language)

**TESEO:** search on June 15, 2011; 75 references.

“Riesgo de caídas”

(search in title and abstract)

**OPEN GREY:** search on June 15, 2011; 3 references.

“hospital falls”
